# The Frequency of Epidermal Growth Factor Receptor (*EGFR*) mutations in Iraqi patients with Non-Small Cell Lung Cancer (NSCLC)

**DOI:** 10.31557/APJCP.2021.22.2.591

**Published:** 2021-02

**Authors:** Hanan H Ramadhan, Dhuha F Taaban, Jubran K Hassan

**Affiliations:** 1 *Department of Clinical Laboratory Science, College of Pharmacy, University of Basrah , Basrah, Iraq.*; 2 *Department of Clinical Pharmacy, College of Pharmacy, University of Basrah , Basrah, Iraq. *

**Keywords:** Non - small cell lung cancer (NSCLC), epidermal growth factor receptor mutations

## Abstract

**Objective:**

Non-Small Cell Lung Cancer (NSCLC) Carcinogenesis could be caused by numerous genetic mutations, one of the most common is the mutation in the Epidermal Growth Factor Receptor (*EGFR*) which was used in the advanced stages of the disease as a therapeutic goal. This study aims to estimate the frequency of Epidermal Growth Factor Receptor mutations in Iraqi patients with Non-Small Cell Lung Cancer.

**Methods:**

One hundred thirty-eight patients confirmed with NSCLC have participated in this study, patients were sent for *EGFR* testing by different oncology centers in Iraq. Data and samples were collected. The Mutation was detected using COBAS^®^ DNA Sample Preparation Kit that designed to detect the following mutations:* Exon 19*: deletions and complex mutations;* Exon 21: L861Q* and *L858R; Exon 18* mutation:* G719X (G719A, G719C*, and* G719S*); *Exon 20: S768I, T790M*, and insertions, this kit utilizes the technology of the real time Polymerase Chain Reaction.

**Results:**

This study was included 79 males and 59 females, with a mean age of 60.1±12.4 years. A positive EGFR mutations were found in 38 (27.53%) of samples. *Exon 19* deletions (25/38, 65.8%) and substitution L858R in exon 21 10/38 (26.3%) were the most common mutations. Multiple mutations (*Exon 20* and 19 combined together) were founded in 2/38 (5.3%), and 1/38 (2.6%) *ALK* mutation. Non-significant differences among age groups and gender in the incidence of mutations were found.

**Conclusion:**

The current study represents the first epidemiological study in Iraq to find *EGFR* mutations frequency among NSCLC patients that reveals the incidence rate of 27.53%, which is between the higher prevalence rate in Asian populations and lower rates in western countries. These results explain the genetic differences of NSCLC in the world due to ethnic differences of the population, more studies are needed in Arab countries to study the *EGFR* mutations, find the effect of ethnicity and environmental factors for lung cancer, and the subsequent therapy.

## Introduction

Lung cancer is the most probable cause of cancer incidence and mortality in the world, with more than 2 million new cases and 1.8 million deaths in 2018 (Bray et al., 2018). Non-small cell lung cancer (NSCLC) accounts for 85% of overall lung cancers (Zhang et al., 2016). Unfortunately 70% of NSCLC patients are diagnosed at an advanced stage, therefore they are not competent for the surgery (Zhou et al., 2017). The treatment protocol based on Chemotherapy as the first choice in the treatment (Goldstraw et al., 2011). Although the overall survival was prolonged and the quality of life was improved by chemotherapy, the prognosis is still poor, particularly in advanced NSCLC (Miltiadou et al., 2020). The median overall survival is 1 year and only 3.5% of patient will survive 5 years after diagnosis(Cataldo et al., 2011). In 2013, a meta-analysis had founded that, the carcinogenesis in NSCLC can be caused by different mutations, one of the most frequent is the mutation in the epidermal growth factor receptor (*EGFR*) (Dearden et al., 2013). The activation of EGFR achieved by binding of its ligands, somatic mutations in these receptors will cause uncontrolled cell division and proliferation by constant receptor activation (Izadian et al., 2017; Benbrahim et al., 2018) . 

The EGFR mutations consider a potential therapeutic target, which is more frequent in adenocarcinomas (ADC) patients and less common in squamous cell carcinoma (SCC) (Yatabe et al., 2015). These mutations are more common among women and non-smokers (Kota et al., 2015). The EGFR mutation rates have been variable according to ethinicty with higher frequency in Asian as compared with western lung cancer patients (Midha et al., 2015; Yatabe et al., 2015). The EGFR mutations frequency in Asians range from 27% to 60% while in European 8% to 13%, the frequency in Africans reaches to 12%, 16% reported in white Americans, and 20% to 25% in India (Palacio et al., 2019). Unfortunately, there are a limited data regarding EGFR mutations in the middle east (Benbrahim et al., 2018), with the absence of frequency determination in Iraq.

In lung cancer, the deletion mutation in exon 19 and point mutation in exon 21 (L858R) represent 90% of *EGFR* mutations. whereas the *T790M* mutations are of low frequency (Arrieta et al., 2015). The active* EGFR *mutations in NSCLC patients exhibit Progression-Free Survival (PFS) to be longer and Objective Remission Rate (ORR) would be better by using EGFR Tyrosine Kinase Inhibitors (TKI) therapy, it also improves life quality, and decrease the side effects related to the treatment when compared with chemotherapy receiving patients (Yatabe et al., 2015). In NSCLC patients several guidelines strongly recommended the testing for *EGFR* mutations and suggested early EGFR-TKIs therapy in patients harboring it (Zhou et al., 2017). Thus, there is a great interest to determine the frequency of *EGFR* mutations. This study aims to estimate the frequency of *EGFR* mutations in Iraq.

## Materials and Methods

*Patients*

This retrospective study included 158 participants with confirmed NSCLC, who have been referred for EGFR testing from different oncology centers throughout Iraq between September 2018 and February 2019. Written informed consent was supplied to all participated members in the current study. The ethics committee in the college of pharmacy/ University of Basrah approved this study. About 20 samples were excluded because of the small sample size, the remaining is 138 samples. Patients’ Demographic data include age, gender, and smoking habits were recorded. No other pathological or clinical information was available since the samples were collected from different cities in the country for this analysis.

Samples were obtained by small Tru-cut biopsy, and the isolated DNA was obtained from the Formalin Fixed Paraffin Embedded (FFPE) tissues.

*Extraction of DNA and Mutational analysis*

Biopsies were collected by Tru-cut biopsy, then the paraffin embedded sections were prepared for DNA extraction process. The DNA Sample Preparation Kit from cobas^®^, is used for DNA extraction from (5-μm) section of formalin fixed paraffin embedded tumors (FFPET) specimens, by preparing the specimen manually depend on the binding of nucleic acid to the glass fibers. The amount of DNA is determined by spectrophotometer, and then a certain concentration was adjusted to be used in the amplification/detection mixture. The cobas z 480 analyzer amplified and detected the target DNA in cobas^® ^EGFR Test using already provided reagents for the amplification - detection. The cobas^® ^EGFR Test is used to perform the *EGFR* mutational analysis, The following mutations can be detected by this kit: Exon 19: deletions and complex mutations; *Exon 21: L861Q* and *L858R*; *Exon 18* mutation:* G719X (G719A, G719C*, and *G719S*); *Exon 20: S768I, T790M*, and insertions, this kit utilizes the technology of the real time Polymerase Chain Reaction (PCR), the target DNA amplification and detection by PCR using fluorescent dyes labeled complementary primer pairs and oligonucleotide probes.

*Statistical analysis*

The data were statistically analyzed by using statistical package for social sciences (SPSS) version 19.0 for windows software. The results of the study are expressed as the mean±standard deviation (SD), The differences between groups were performed by using the chi-square test. The statistical significance was considered at a p-value of less than 0.05. 

## Results

The population of the study consists of 158 tumor samples, which were received for analysis of *EGFR *mutations, 20 samples with a small sample size were excluded. In all patients, the most common *EGFR* mutations *(exon 19* deletion, *exon 20 T790M* mutation, and *exon 21 L858R* point mutation) were analyzed. Out of 138 studied cases, 79 (57.2%) were males and 59 (42.8%) were female (Table 1). The mean age of the participant was 60.1±12.4 years. A positive *EGFR *mutations were detected in 38 patients (27.53%), the most frequent mutation was a deletion in *exon 19* (25 of 38 patients, 65.8%), while only 10 of 38 (26.3%) patients had a substitution L858R in exon 21. Multiple mutations (*Exon 19* and *Exon 20* mutations combined together) have been found in 2 of 38 patients (5.3%), table 1 shows the distribution of *EGFR* mutations. Only 1 case showed another type of mutation, *ALK* mutation (2.6%). There was no evidence of mutations that could be found in 100 patients (72.47%) (Table 1).

Table 2 showed that *EGFR* mutations were detected in 16 of 59 females (27.1%), and 22 of 79 males (27.8%), with no statistically significant differences, have been found between genders (p=0.924), among age groups. (p=0.816) and different regions in Iraq (p=0.892).

A subgroup analysis was also conducted to detect whether the distribution of* EGFR* mutation types was influenced by gender or age. This study revealed that there were no significant differences were observed in the distribution of types of *EGFR* mutation by sex and age. As seen in (Tables 3 and 4).

**Table 1:** Demographic Data of Patients

	study group N=138	p- value
Age ( years)	60.1 ± 12.4	0.562
35-50	37(26.8%)	0.009
50-65	38(27.5%)	
>65	63(45.7%)	
Gender		
Male	79 (57.2%)	0.0887
Female	59 (42.8%)	
% Mutation		
+ve mutation	38 (27.53%)	<0.0001
-ve mutation	100 (72.47%)	
Mutation distribution N=38		
L858R	10 (26.3%)	<0.0001
Ex19Del	25 (65.8%)	
Ex19Del, T790M	2 (5.3%)	
ALK Mutation	1 (2.6%)	

p value <0.05 considered as significant values

**Figure 1:**
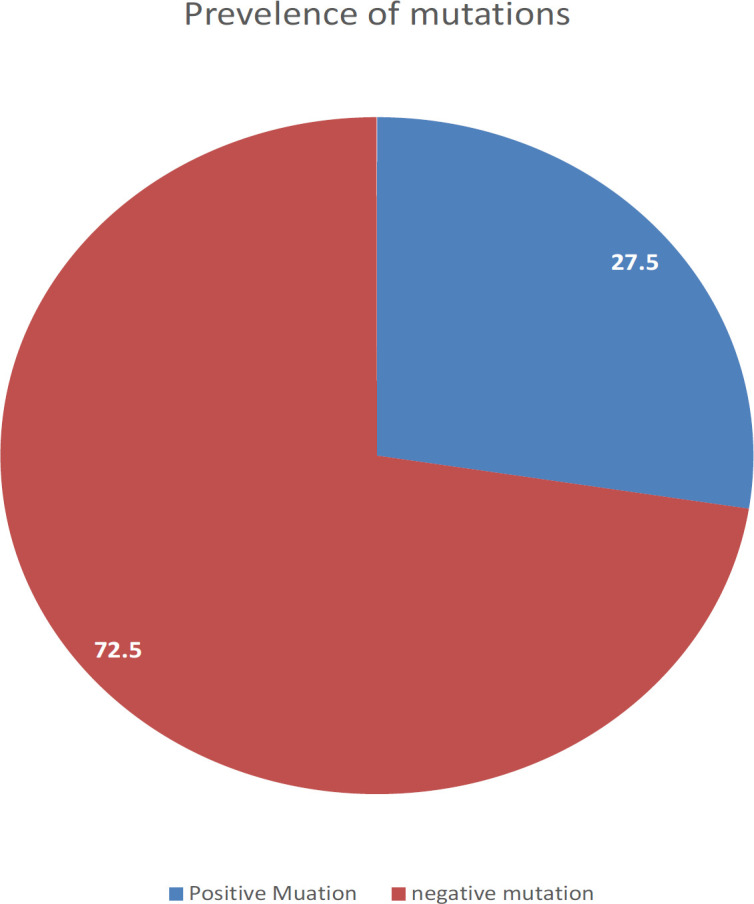
The Prevalence of *EGFR* Mutations in Iraq

**Figure 2:**
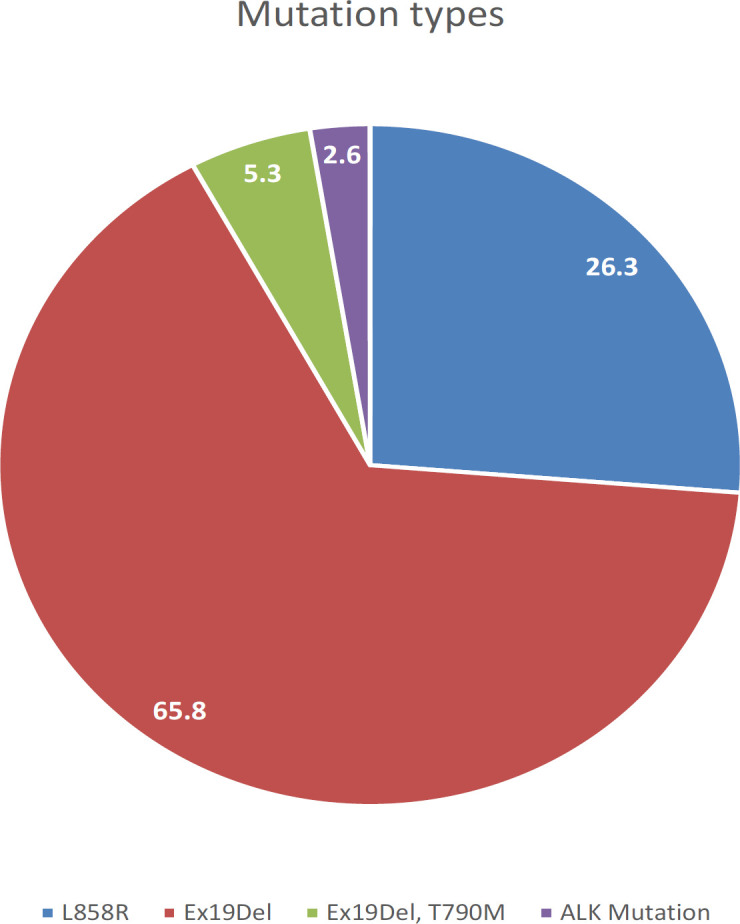
The Distribution of Mutations by Exon

**Table 2:** Mutation Prevalence According to Gender and Age

Parameter	Total	Positive	Negative	P values
Mutation prevalence according Gender				
Male	79	22 (27.8%)	57 (72.2%)	0.924
Female	59	16 (27.1%)	43 (72.9%)	
Mutation prevalence according Age				
35-50	37	11 (29.7%)	26 (70.3%)	0.816
50 -65	38	9 (23.7%)	29 (76.3%)	
≥65	63	18 (47.4%)		
Mutation prevalence according to Regions				
Babil	11	4 (36.4%)	7 (63.6%)	0.892
Baghdad	44	11 (25%)	33 (75%)	
Basra	21	7 (33.3%)	14 (66.7%)	
AL-Diwania	4	1 (25%)	3 (75%)	
Erbil	4	2 (50%)	2 (50%)	
Kirkuk	7	3 (42.9%)	4 (57.1%)	
Karbala	9	3 (33.3%)	6 (66.7%)	
Najaf	8	1 (12.5%)	7 (87.5%)	
Sulaimanyah	13	2 (15.4%)	11 (84.6%)	
Thi-Qar	10	4 (40%)	6 (60%)	

p-values <0.05 considered as significant values

**Table 3:** Positive Mutation Details According to Gender

Parameter	Gender		P values
	Male	female	
L858R	6 (27.3%)	4 (25%)	0.877
Ex19Del	13 (59.1%)	12 (75%)	0.314
Ex19Del, T790M	2 (9.1%)	0 (0%)	0.221
ALK Mutation	1 (4.5%)	0 (0%)	0.394

p value <0.05 considered as significant values

**Table 4:** Positive Mutation Details According to Age

Type of Mutation	35-50 years N= 11	50 -65 years N=9	≥65 years N=18	P values
L858R	4 (36.4%)	2 (22.2%)	4 (22.2%)	0.668
Ex19Del	6 (54.5%)	6 (66.7%)	13 (72.2%)	0.621
Ex19Del, T790M	1 (9.1%)	0 (0%)	1 (5.6%)	0.661
ALK Mutation	0 (0%)	1 (11.1%)	0 (0%)	0.214
P value	0.1778	0.097	0.0015	

p value <0.05 considered as significant values

## Discussion

The analysis of EGFR mutations is important for the diagnosis and treatment decision in NSCLC, especially adenocarcinoma, as EGFR mutations are a potential predictive biomarker for the EGFR-TKI therapy response (Zhou and Christiani, 2011; Shi et al., 2015). The overall prognosis can be improved by EGFR mutations testing and using the EGFR-TKI therapy early, as the chemotherapy has limited efficacy in NSCLC patients (Zhang et al., 2016). In the present study, 138 patient samples from different cities were analyzed to estimate the EGFR mutations frequency in Iraq. This is the first study in Iraq, so the results can be used as a reference for research in the future.

In the current study, the EGFR mutations were detected in 27.53% of NSCLC patients. Despite, the prevalence rate was higher than that reported in the Saudi population and Lebanese population (Al-Kuraya et al., 2006; Fakhruddin et al., 2014; Kattan et al., 2015; Naderi et al., 2015), and higher than the prevalence rate reported by Tfayli et al in 2017 published study (Tfayli et al., 2017), this finding was still consistent with EGFR mutations frequency in the Middle East region, that ranging from 2.9% to 28.7% (Errihani et al., 2013; Jazieh et al., 2015; Zaki et al., 2015; Benbrahim et al., 2018), and lower than Asian countries that reached 45.5% (Yatabe et al., 2015; Tfayli et al., 2019). However, a review of 19 European studies by Szumera-Cieckiewicz et al, reported frequencies from European countries between (2.6% and 39%) in Italy and Germany, respectively (Szumera-Cieckiewicz et al., 2013). In Latin America, the updated research of 5738 cases in 2015, revealed that the frequency of EGFR mutations varies from 14.4% in Argentina, to 51.1% in Peru (Arrieta et al., 2015). Leary et al, 2012, revealed the incidence of EGFR mutations in UK was 11% (Leary et al., 2012). Another study from turkey demonstrated a frequency of EGFR mutations was 30% (A et al., 2018). Similarly, a study in the US, conducted that the EGFR mutations prevalence rate was 20% (Reinersman et al., 2011), and Michele L. Cote et al reported the frequency of 13.9% in African-Americans (Cote et al., 2011). These differences in prevalence rate have resulted from different geographical regions, the ethnicity of these populations (Arrieta et al., 2015; Zhou et al., 2017; Benbrahim et al., 2018), and also may be due to differences in the testing methods (Benbrahim et al., 2018).

In the current study, deletion in exon 19 was the most frequent type of EGFR mutations (65.8%) followed by L858R (26.3%) of all mutations, this is similar to studies in the East Asian countries (Liam et al., 2013; Shi et al., 2014; Lee et al., 2015; Shi et al., 2015). And is comparable to the study published in 2017, that revealed a deletion in exon 19 represents 78.1%, followed by exon 21 mutation 21.9% of all mutations (Tfayli et al., 2017). In contrast, Berois et al, reports that the most common mutations are rare mutations and only 40% of all mutations is the mutations in exon 19 and exon 21 (Berois et al., 2017). Liu et al, reported that (80-90%) of all mutations are the deletion mutation in exon 19 and missense mutation L858R in exon 21 together, these would be the best predictors for TKIs response, with better outcomes reported for exon 19 deletions than point mutation L858R (Liu et al., 2016). Whereas, the T790M mutation found as a complex mutation in 2 patients (5.3%) of all mutations. However, this result is higher than the incidence of T790M mutation reported by Zhou et al, and Shi et al (Shi et al., 2015; Zhou et al., 2017). These differences in the prevalence of T790M EGFR mutation may be due to variations in the technique and population (Zhou et al., 2017).

The response to treatment, prognosis, and survival rates varies with the mutation type, so the study of mutations type is of particular clinical importance. These findings have been supported by several studies, Jackman et al., 2006 and Li at al., 2011 who reported that better prognosis and long survival were associated with the mutation in exon 19 and 21, whereas poor prognosis was correlated with a mutation in exon 20 (Jackman et al., 2006; Li et al., 2011). Accordingly response rate to the treatment with TKIs were found to be improved in exon 19 and 21 mutations while exon 20 was showed resistance to the therapy (Unal et al., 2013). Longer survival rates were detected in patients with exon 19 deletions treated with TKIs (Cote et al., 2011), while short progression free survival has been exhibited in NSCLC patient with T790M mutation received EGFR-TKI treatment (Shi et al., 2015).

The previous Asian studies conducted a higher prevalence rate of EGFR mutations in the female gender. Leary et al., (2012) and Demiray et al., (2018) are in line with the Asian results and consider the female gender as one of the predictors of EGFR mutations (Leary et al., 2012; A et al., 2018). Another study published in 2015, from three countries KSA, United Arab Emirates and Qatar demonstrated the significant association of the EGFR prevalence rate with the female gender (Jazieh et al., 2015). Interestingly, in our study, the EGFR mutations were found in 27.1% females and 27.8% male patients, and the effect of gender was not significant. This result is in agreement with the published study by Unal et al, who found that, although the EGFR mutation rate is higher in females, it does not reach a statistically significant level (Unal et al., 2013). 

This study did not find any significant difference in the rate of mutations across different regions in Iraq, that have been suggested, the highest EGFR mutations rate was in Baghdad 13 (34.2%), followed by Basra 7 (18.4), and the lowest rate was in Erbil and Al-Diwania 1(2.6%). A larger scale study with an extended period of time required to study the differences in mutation rate among different geographic regions. 
